# Genetic enhancement of neuropathic and inflammatory pain by forebrain upregulation of CREB-mediated transcription

**DOI:** 10.1186/1744-8069-8-90

**Published:** 2012-12-31

**Authors:** Giannina Descalzi, Hotaka Fukushima, Akinobu Suzuki, Satoshi Kida, Min Zhuo

**Affiliations:** 1 Department of Physiology, Faculty of Medicine, University of Toronto, 1 King's College Circle; University of Toronto Center for the study of Pain, Toronto, Ontario, M5S 1A8, Canada; 2 Center for Neuron and Disease, Frontier Institute of Science and Technology, Xi’an Jiaotong University, 28 Xianning West Road, Xian, Shaanxi, 710049, China; 3 Department of Bioscience, Faculty of Applied Bioscience, Tokyo University of Agriculture, Tokyo, Japan

## Abstract

CREB has been reported to be activated by injury and is commonly used as marker for pain-related plasticity changes in somatosensory pathways, including spinal dorsal horn neurons and the anterior cingulate cortex (ACC). However no evidence has been reported to support the direct role of activated CREB in injury-related behavioral sensitization (or allodynia). Here we report that genetic enhancement of CREB-mediated transcription selectively in forebrain areas enhanced behavioral responses to non-noxious stimuli after chronic inflammation (CFA model) or nerve injury. In contrast, behavioral acute responses to peripheral subcutaneous injection of formalin did not show any significant difference. Furthermore, acute pain responses to noxious thermal stimuli were also not affected. Our results thus provide direct evidence that cortical CREB-mediated transcription contributes to behavioral allodynia in animal models of chronic inflammatory or neuropathic pain.

## Background

Activity-dependent gene expression is important for long term changes in synaptic transmission [1,2]. Increases in intracellular Ca^2+^ concentrations activate various intracellular signaling cascades, including cAMP and Ca^2+^ calmodulin (CaM) dependent protein kinase pathways [3]. These pathways in turn lead to the phosphorylation of the transcription factor cAMP responsive element binding protein (CREB) at the serine 133 site [4]. CREB is a major transcription factor that plays a central role in the formation of long-term memory [5-10]. Accordingly, genetic enhancement of forebrain CREB corresponds with enhancements in long term memory and late-phase long term potentiation (L-LTP) in the CA1 region of the hippocampus [11]; whereas genetic inhibition of CREB in the nucleus accumbens increases the rewarding efficacy of cocaine [12].

Peripheral injury triggers long term potentiation of transmission in cortical ACC synapses [13-15]. Although injury-induced activation of CREB (phosphorylation) in cortical areas has been reported [7], no study has shown direct evidence that CREB-mediated transcription in the cortex actually contributes to behavioral sensitization. To address this question, we used transgenic mice expressing dominant active CREB mutant in the forebrain (Y134F) driven by the αCaMKII promoter [11], which is expressed predominantly in forebrain regions, including cirtical areas, amygdala, and hippocampus [16]. These mice show increased CREB activity to sensory stimuli, and exhibit enhancements in long term memory and L-LTP in the CA1 region of the hippocampus [11]. In this short report, we present evidence that enhanced CREB activity in the forebrain leads to the enhancement of behavioral responses to non-noxious stimuli after injury.

**Figure 1:**
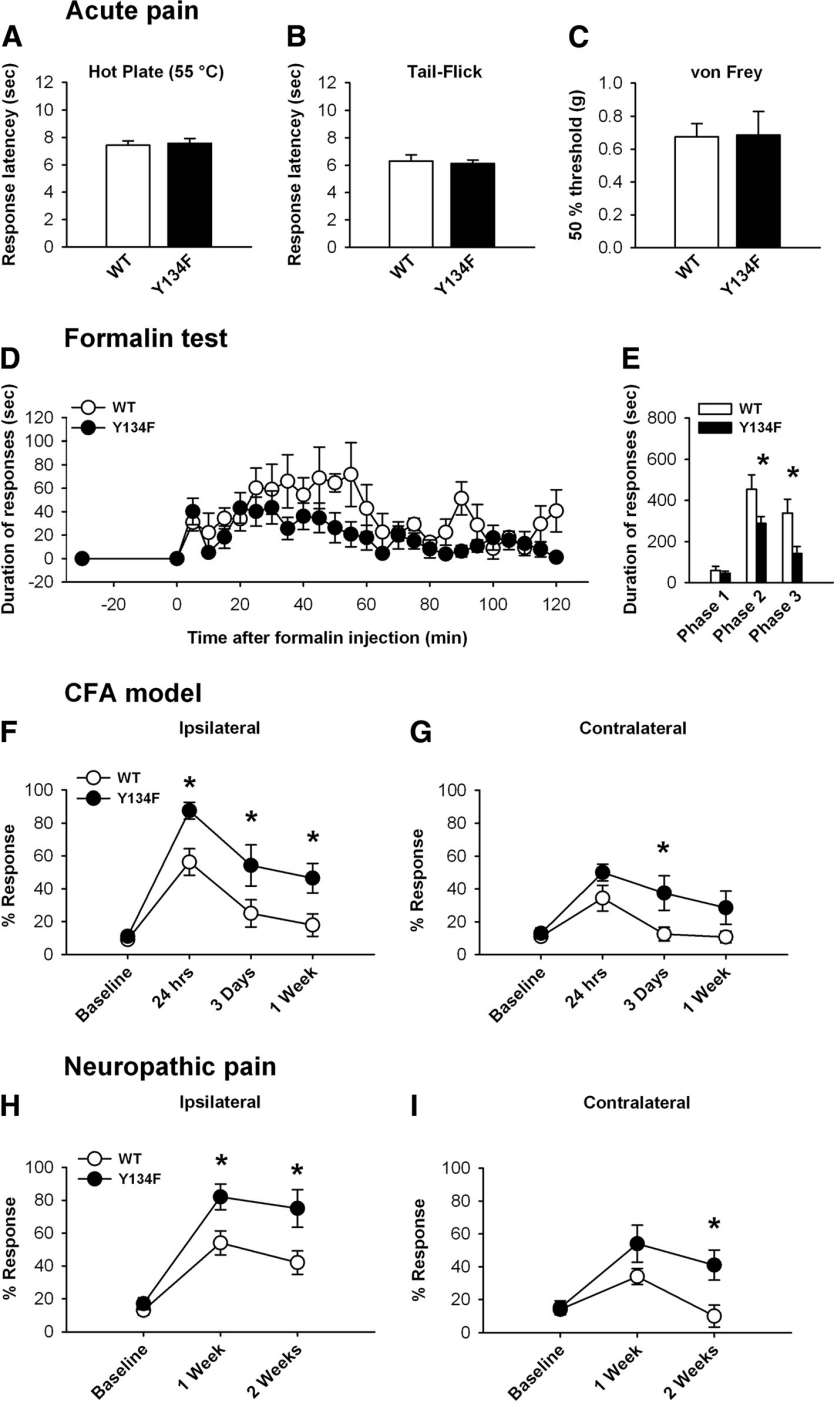
**Behavioral assessment of acute, inflammatory, and neuropathic pain. A**) Acute nociception was unaltered by forebrain CREB overexpression. Response latencies in the hot plate and **B**) tail flick tests were similar between groups. **C**) 50% mechanical threshold levels were indistinguishable between groups. **D**) Formalin test, Y134F mice show significantly less licking responses than WT mice (*F*_(1, 22)_ = 12.68, *P* = 0.004). **E**) A significant interaction was detected, whereby Y134F mice only showed decreases in Phase 2 (10 – 60 min) and 3 (60 – 120 min), but not Phase 1 (0 – 10 min) of the formalin test. Phase 1 corresponds with acute inflammatory pain, whereas Phase 2 and 3 represent more tonic states of pain. **F**) In the CFA model of chronic inflammatory pain, Y134F mice showed a significant enhancement in CFA induced mechanical allodynia up to 1 Week post CFA application (*F*_(1, 18)_ = 21.67, *P* = 0.003). **G**) Enhanced allodynia was also seen in the uninjected paw. **H**) In the neuropathic pain model, Y134F mice showed a significant enhancement in mechanical allodynia up to 2 weeks post surgery (*F*_(1, 24)_ = 43.61, *P* < 0.001). **I**) Neuropathic pain also produced a significant enhancement in the uninjected paw at 2 weeks.

In order to determine if forebrain overexpression could affect acute nociception, we first exposed both groups of mice to the hot plate (55°C) and tail flick tests of thermal nociception. Both groups of mice showed similar response latencies to the hot plate (WT: 7 ± 1 sec, n = 7; Y134F: 8 ± 1 sec, n = 7; Figure 1A) and tail flick tests (WT: 6 ± 1 sec, n = 7; Y134F: 6 ± 1 sec, n = 7; Figure 1B). Acute pain is physiological, with an obvious advantageous survival purpose. This form of pain is insensitive to genetic or pharmacological inhibition of Ca^2+^ calmodulin dependent intracellular pathways [7,17]. Accordingly, both groups of mice also showed similar 50% mechanical response thresholds (WT: 0.7 ± 0.1 g, n = 7; Y134F: 0.7 ± 0.1 g, n = 7; Figure 1C). These results indicate that CREB overexpression in the forebrain does not alter basal nociceptive thresholds.

We next sought to determine the effects of forebrain CREB overexpression on acute inflammatory pain by measuring behavioral (licking) responses to inflammation brought on by intradermal injections of formalin (5%) to the hindpaw [18]. Transgenic mice with forebrain NMDA receptor subtype NR2B overexpression show a robust enhancement of acute inflammatory pain [18]; whereas transgenic mice with a genetic deletion of adenylyl cyclase type 1 (AC1) show reduced behavioral nociceptive responses to peripheral injection of formalin [17,19]. Within the first 10 min after formalin injection, behavioral responses were undistinguishable between WT and Y134F mice (Figure 1D). In the subsequent phases of the test, however, Y134F mice showed a marked reduction in responses, which lasted well into 2 hrs post injection. Indeed, a repeated measures two-way ANOVA revealed a significant main difference in licking time between WT and Y134F mice, and showed a significant interaction between group and time, whereby Y134F mice showed significantly less licking behavior only during phases 2 and 3, but not 1 (Phase 1: WT: 59 ± 20 sec, n = 6; Y134F 45 ± 10 sec, n = 7; *P* = 0.8; Phase 2: WT: 454 ± 71 sec, n = 6; Y134F 288 ± 34 sec, n = 7; *P* < 0.001; Phase 3: WT: 339 ± 68 sec, n = 6; Y134F 141 ± 34 sec, n = 7; *P* < 0.001; Figure 1E). The lack of difference during phase one is consistent with the observation that acute behavioral responses to noxious stimuli were not affected in the same mice. In the later stages of the test however (Phase 2 and 3), we saw a marked reduction in licking behavior. These results however differ from previous reports in mice with NR2B forebrain overexpression, indicating that genetic manipulation at downstream signaling targets may cause different phenotypes [18]. Future studies are needed to investigate the exact mechanism. This finding also raises the possibility that cortical CREB activity may not directly contribute to behavioral responses in cases of acute inflammation.

Next, we assessed mechanical sensitization in a chronic inflammatory pain model using complete Freund’s adjuvant (CFA) injected into the hind paw. We measured mechanical allodynia in mice by quantifying paw withdrawal behavior in response to applications of an innocuous mechanical stimulus, which under normal conditions (baseline) yields very few responses [18]. Y134F mice showed robust enhancements of mechanical allodynia (Figure 1F), which was apparent at 24 h (WT: 56 ± 8%, n = 4; Y134F 88 ± 5%, n = 4; *P* = 0.007), 3 days (WT: 25 ± 8%, n = 4; Y134F 54 ± 13%, n = 4; *P* = 0.01), and 1 week after CFA injection (WT: 18 ± 7%, n = 4; Y134F 46 ± 9%, n = 4; *P* = 0.01). In the contralateral paw, differences were only detected at 3 days post injection (WT: 13 ± 4%, n = 4; Y134F 38 ± 11%, n = 4; *P* = 0.01; Figure 1G).

Nerve injury (neuropathic pain model) activates Ca^2+^ calmodulin dependent pathways within the anterior cingulate cortex (ACC) [7,19], a forebrain structure involved in the affective component of pain [20,21]. We next compared behavioral responses in WT and CREB-Y134F mice after exposing both groups to ligation of the common peroneal nerve, which induces robust chronic pain in mice lasting weeks [22]. Although no differences could be detected at baseline, a repeated measures two-way ANOVA revealed a significant difference of mechanical allodynia between groups (Figure 1H). Post-hoc analyses further showed that Y134F mice present a significant enhancement in mechanical allodynia at 1 and 2 weeks after nerve ligation surgery (1 week: WT: 54 ± 7%, n = 7; Y134F 82 ± 8%, n = 7; *P* < 0.001), (2 weeks: WT: 42 ± 7%, n = 6; Y134F 75 ± 11%, n = 7; *P* < 0.001). Interestingly, Y134F mice also showed enhanced allodynia in the contralateral (non-injured) hindpaw, which remained significantly higher after 2 weeks (1 week: WT: 34 ± 5%, n = 7; Y134F 54 ± 11%, n = 7; *P* = 0.3) (2 weeks: WT: 10 ± 7%, n = 6; Y134F 41 ± 10%, n = 7; *P* = 0.005; Figure 1I).

In conclusion, we report here for the first time, to our knowledge, that enhancement of CREB activity within forebrain neurons potentiates behavioral responses to sensory stimuli in animal models of chronic inflammatory pain and neuropathic pain. This is supported by previous reports that synaptic potentiation (or called LTP) is enhanced in the regions of the hippocampus [11] and the ACC (Chen et al., unpublished data). In contrast, observations that basal excitatory synaptic transmission is not affected in the same transgenic mice supports our current findings that acute responses to physiological noxious thermal and mechanical stimuli were unaffected. Accordingly, forebrain CREB overexpression does not affect the first phase of the formalin test, during which behavioral responses represent direct activation of nociceptive pathways. In contrast, reduction of responses were observed in the later phases (2 and 3), which are thought to be mediated by mechanisms involved in central sensitization of nociceptive transmission [23]. These reductions were surprising, and caution should therefore be exercised before using CREB as a marker for acute inflammatory pain. For example, previous observations in the spinal cord have shown formalin-induced CREB phosphorylation in ipsilateral and contralateral dorsal root ganglion (DRG) neurons that reached peak expression at 10 min [23], whereas behavioral responses to formalin injections last beyond 1 hr.

Chronic pain produced through peripheral inflammation or nerve injury corresponds with potentiation of excitatory transmission in ACC synapses [13,14,24], which is partly induced through increases in postsynaptic AMPA receptor GluA1 subunits [13]. Gene expression is important for long term changes in synaptic transmission [1,2], and CREB has been implicated in various events that are known to correspond with changes in postsynaptic receptors including fear learning [8,9,11] and drug addiction [25]. Chronic pain requires the activation of cAMP and Ca^2+^ -CaM dependent protein kinase pathways, and the disruption of these pathways reduces chronic pain [7,26]. As chronic pain [13] and fear learning [27] have been shown to correspond with increases in postsynaptic AMPA GluA1 receptors within the ACC, it is likely that CREB is involved in the pain induced, Ca^2+^ CaM dependent, upregulation of postsynaptic AMPA GluA1 receptors, and thus contributes to enhancements in excitatory synaptic transmission within the ACC. The ACC receives robust projections from the thalamus and in turn also projects to thalamic and spinal pathways [28,29]. Forebrain overexpression of CREB can thus facilitate increases in excitatory transmission within the ACC, and enhance top-down descending facilitation of spinal sensory transmission [30-32]. It is important to note however, that as the mice used in this study express the dominant active CREB mutant transgene in a number of forebrain structures, other forebrain areas may also be involved, such as the insular cortex, amygdala, and hippocampus. Future studies are needed to investigate experience-induced synaptic and structural changes in the transgenic mice.

## Methods

### Animals

Adult (8–12 month) transgenic mice Y134F line C, expressing dominant active CREB in the forebrain, and age matched WT littermates were used for all studies as reported previously [11]. Genotypes were identified by PCR analysis of genomic DNA extracted from mouse ear tissue. All mice were housed under a 12 h light/dark cycle with food and water provided *ad libitum*. All mouse protocols are in accordance with National Institutes of Health guidelines and approved by the Animal Care and Use Committee of University of Toronto.

### Behavioral experiments

Acute pain assessment was performed as published previously [18]. Briefly, we determined the latency of behavioral responses to placement on a thermal hot plate (55°C) (Columbus Instruments, Columbus, OH), and the latency for the spinal nociceptive tail-flick reflex, evoked by radiant heat applied to the underside of the tail (Columbus Instruments, Columbus, OH). All tests were performed blind to genotype. Fifty percent mechanical threshold was assessed with a set of von Frey filaments (Stoelting, Wood Dale, Illinois) using the up-and-down method [33]. Briefly, mice were placed in plexiglass containers with elevated wire mesh flooring, and were allowed to acclimate for 30 min before testing. A threshold stimulus was determined by observing animal hind paw withdrawal upon application of a von Frey filament; positive responses included prolonged hind paw withdrawal, or licking or biting of the hind paw. Mechanical allodynia was measured as described previously [18], and was assessed with the 0.4 mN (No. 2.44) von Frey filament, previously observed to produce minimal hind paw withdrawal in untreated mice [18]. Positive responses included licking, biting, and prolonged withdrawal of the hindpaw. Experiments consisted of 10 trials, with 10 min inter-trial intervals. All observations were performed blind.

### Inflammatory pain models

Formalin (5%, 10 μl; Sigma-Aldrich) or complete Freund’s adjuvant (CFA, 50%, 10 μl; Sigma-Aldrich) was injected subcutaneously into the dorsal side of the left hind paw as reported previously [18]. In the formalin test, the total time spent licking or biting the injected hind paw was recorded for each five-minute intervals for two hours post injection. In the CFA model, mechanical sensitivity was assessed.

### Neuropathic pain model

The neuropathic pain model consisted of ligation of the common peroneal nerve (CPN) and was performed as previously described [13,22]. Briefly, mice were anesthetized by inhaled isofluorane (1-3%). A 1 cm skin incision, from the fibular head to the lateral side of the ankle joint, was made, followed by an incision of the subcutaneous tissue. A vertical incision was made of the white fascia, and the posterior muscles were pulled laterally to expose the CPN, which was ligated with 5–0 chromic gut sutures (Ethicon). Skin was sutured with sterile 5–0 silk.

### Data analysis

Results were expressed as mean ± s.e.m. Unpaired student t-tests and two-way repeated Analyses of Variance (ANOVA) were performed, with the Holme-Sidak test for multiple comparisons performed post-hoc if significant differences were observed. In all cases *P* < 0.05 was considered statistically significant.

## Competing interests

The authors declare that they have no competing interests.

## **Authors’ contributions**

MZ designed experiments, GD designed and performed experiments. GD, HT, AS, SK and MZ wrote manuscript. All authors read and approved final manuscript.
